# Erratum to “Suffering from Cerebral Small Vessel Disease with and without Metabolic Syndrome”

**DOI:** 10.1515/med-2021-0006

**Published:** 2020-11-27

**Authors:** Matić TB, Toncev G, Gavrilović A, Aleksić D

**Affiliations:** University of Kragujevac, Serbia, Faculty of Medical Sciences, Department of Neurology and Clinical Center, Kragujevac, Serbia

In the published article “Matić TB, Toncev G, Gavrilović A, Aleksić D. Suffering from Cerebral Small Vessel Disease with and without Metabolic Syndrome. Open Med (Wars). 2019 Jun 11;14: 479–84. doi: 10.1515/med-2019-0051. PMID: 31231684; PMCID: PMC6572407” the affiliation of the first and corresponding author – Dr. Tatjana Bošković Matić is incorrect. Dr Tatjana Bošković Matić is affiliated to University of Kragujevac, Faculty of Medical Science, which was missed by mistake in the publication itself.

The correct affiliation of Dr Tatjana Bošković Matić is as follows:

Tatjana Bošković Matić, University of Kragujevac, Serbia, Faculty of Medical Sciences, Department of Neurology and Clinical Center, Kragujevac, Serbia, tel: +381 64 112 96 21, e-mail: stmatic@ptt.rs

